# Omicron SARS-CoV-2 infection management and outcomes in patients with hematologic disease and recipients of cell therapy

**DOI:** 10.3389/fonc.2024.1389345

**Published:** 2024-06-19

**Authors:** José Luis Piñana, Lourdes Vazquez, Inmaculada Heras, Tommaso Francesco Aiello, Lucia López-Corral, Ignacio Arroyo, Eva Soler-Espejo, Irene García-Cadenas, Valentín Garcia-Gutierrez, Cristina Aroca, Pedro Chorao, María T. Olave, Javier Lopez-Jimenez, Marina Acera Gómez, Elena Arellano, Marian Cuesta-Casas, Alejandro Avendaño-Pita, Clara González-Santillana, José Ángel Hernández-Rivas, Alicia Roldán-Pérez, Mireia Mico-Cerdá, Manuel Guerreiro, Julia Morell, Paula Rodriguez-Galvez, Jorge Labrador, Diana Campos, Ángel Cedillo, Carolina Garcia Vidal, Rodrigo Martino, Carlos Solano

**Affiliations:** ^1^ Hematology Department, Hospital Clínico Universitario de Valencia, Valencia, Spain; ^2^ INCLIVA Biomedical Research Institute, Valencia, Spain; ^3^ Hematology Department, University Hospital of Salamanca (HUS/IBSAL), CIBERONC and Cancer Research Institute of Salamanca-IBMCC (USAL-CSIC), Salamanca, Spain; ^4^ Hematology Division, Hospital Morales Meseguer, Murcia, Spain; ^5^ Infectious Disease Division, Hospital Clinic, Barcelona, Spain; ^6^ Hematology Division, Hospital de la Santa Creu i Sant Pau, Barcelona, Spain; ^7^ Hematology Division, Hospital Ramon y Cajal, Madrid, Spain; ^8^ Hematology Division, Hospital Universitario y Politécnico La Fe, Valencia, Spain; ^9^ Hematology Division, Hospital Clínico Universitario Lozano Blesa, IIS Aragon, Zaragoza, Spain; ^10^ Hematology Division, Hospital Universitario Virgen Macarena, Sevilla, Spain; ^11^ Hematology Division, Hospital Regional Universitario Carlos Haya, Malaga, Spain; ^12^ Hematology Division, Hospital de Fuenlabrada, Madrid, Spain; ^13^ Hematology Division, Hospital Universitario Infanta Leonor, Madrid, Spain; ^14^ Hematology Division, Hospital Infanta Sofia, Madrid, Spain; ^15^ Research unit, Hospital Universitario de Burgos, Burgos, Spain; ^16^ Institute of Experimental and Clinical Pharmacology and Toxicology, Center for Brain, Behavior and Metabolism (CBBM), University of Lübeck, Lübeck, Germany; ^17^ Hematopoietic Stem Cell Transplantation and Cell Therapy Group (GETH-TC) office, Madrid, Spain; ^18^ Department of Medicine, School of Medicine. University of Valencia, Valencia, Spain

**Keywords:** SARS-CoV-2, hematologic disease, immunocompromised, risk factors, COVID - 19

## Abstract

**Introduction:**

Scarce real-life data exists for COVID-19 management in hematologic disease (HD) patients in the Omicron era.

**Purpose:**

To assess the current clinical management and outcome of SARS-CoV-2 infection diagnosed, identify the risk factors for severe outcomes according to the HD characteristics and cell therapy procedures in a real-world setting.

**Methods:**

A retrospective observational registry led by the Spanish Transplant Group (GETH-TC) with 692 consecutive patients with HD from December 2021 to May 2023 was analyzed.

**Results:**

Nearly one-third of patients (31%) remained untreated and presented low COVID-19-related mortality (0.9%). Nirmatrelvir/ritonavir was used mainly in mild COVID-19 cases in the outpatient setting (32%) with a low mortality (1%), while treatment with remdesivir was preferentially administered in moderate-to-severe SARS-CoV-2 infection cases during hospitalization (35%) with a mortality rate of 8.6%. The hospital admission rate was 23%, while 18% developed pneumonia. COVID-19-related mortality in admitted patients was 14%. Older age, autologous hematopoietic stem cell transplantation (SCT), chimeric antigen receptor T-cell therapy, corticosteroids and incomplete vaccination were factors independently associated with COVID-19 severity and significantly related with higher rates of hospital admission and pneumonia. Incomplete vaccination status, treatment with prior anti-CD20 monoclonal antibodies, and comorbid cardiomyopathy were identified as independent risk factors for COVID-19 mortality.

**Conclusions:**

The results support that, albeit to a lower extent, COVID-19 in the Omicron era remains a significant problem in HD patients. Complete vaccination (3 doses) should be prioritized in these immunocompromised patients. The identified risk factors may help to improve COVID-19 management to decrease the rate of severe disease, ICU admissions and mortality.

## 1 Introduction

Individuals with a weakened immune system are a minority in the population and have been poorly represented in large COVID-19 randomized controlled trials (RCTs) (1, 2). Immunocompromised patients with hematologic diseases (HDs) have a longer duration of SARS-CoV-2 symptoms and infectiousness, shedding viable virus particles up to 4 weeks after testing positive, in contrast to 10–12 days in non-immunocompromised asymptomatic individuals (3, 4). These patients may thereby host a suitable environment for viral mutagenesis (5). Additionally, prior to mass vaccination, the COVID-19 mortality rate reached 30% in patients with HD, and, in those older than 70 years, the rate reached almost 50% (6). These numbers were in striking contrast with the general population, where in the worst moments of the pandemic (June 2020), countries like Italy and Spain presented a mortality rate of 14.5% and 11.7%, respectively, not surpassing 1.9% in the whole world since January 2023 (*data from* ourworldindata.org) (6–8).

Vaccination reduced COVID-19 mortality to less than 2% in HD patients (9). Strong vaccine-induced antibody response in these patients seems critical for protection against both breakthrough infection and severe disease, even with the Omicron SARS-CoV-2 variant (9). However, an impaired response to full SARS-CoV-2 vaccination occurs in 5% to 70% of immunocompromised patients depending on age, disease type, the timing and type of HD treatment. For instance, further vaccination doses are necessary to reach levels of neutralizing antibodies against the virus that are, nonetheless, not comparable to the ones achieved in healthy individuals after booster doses (10–12). Specifically, infection with the Omicron SARS-CoV-2 variant of concern (VOC) still represents a strong threat for in-hospital mortality in poor responders or those not fully vaccinated and older immunocompromised patients (3.5%), although not associated with a risk factor for longer shedding of viable virus (13, 14).

The recommendation for treating SARS-CoV-2 infection in immunocompromised patients is early treatment with antiviral and/or monoclonal antibodies with sustained neutralizing activity in cases with mild-severe COVID-19, but there are few real-life studies on these patients describing the current COVID-19 management strategies and the risk of disease progression, COVID-19 mortality, and virus shedding (15–18). The work of Mikulska and collaborators was pioneer in reporting outcomes of early treatment of SARS-CoV-2 infection in a representative number of patients with HD (13). Treatment for COVID-19 with antivirals correlated with shorter viral shedding, while administration of monoclonal antibodies alone was shown as a predictor of treatment failure (13). However, combination treatment using antiviral and monoclonal antibodies – a triple combination of remdesivir, nirmatrelvir or molnupiravir followed by anti-spike monoclonal antibodies specific for the infecting strain – is feasible and displayed high efficacy in early virological response and 30-day virological and clinical response in small case-series reports (19, 20).

In this scenario, the current real-word study assessed current management, clinical presentation, severity and outcome of SARS-CoV-2 Omicron VOC infections and risk factors for adverse clinical outcome according to the HD characteristics and cell therapy procedures in the post-vaccination era through a retrospective observational registry conducted by the Spanish Hematopoietic Stem Cell Transplantation and Cell Therapy Group (GETH-TC) in a large cohort of patients with HD.

## 2 Methods

### 2.1 Study design and patients

In April 2023, the Infectious Complications Subcommittee (GRUCINI) of the GETH-TC launched a national retrospective multicenter registry to evaluate current management strategies, severity, and outcome of breakthrough SARS-CoV-2 Omicron VOC infection in immunocompromised patients with HD, including recipients of cell therapy. The registry included consecutive adult patients with HD, diagnosed with SARS-CoV-2 infection either by PCR or rapid antigen tests, with clinical symptoms or asymptomatic cases if they had received antiviral therapy between December 27, 2021, to May 30, 2023, in 13 participating Spanish centers. The status of all included patients was updated on July 10, 2023, with the study database.

The local Research Ethics Committee of the Hospital Clínico Universitario de Valencia approved the study (reference code 35.21), which was carried out in accordance with the Declaration of Helsinki and its amendments, and applicable national regulatory requirements. The Research Ethics Committee approved the waiver of informed consent for inclusion in the study.

Data retrospectively collected in this registry included demographic (age, sex) and clinical characteristics of patients, including comorbidity and data related to HD, including underlying malignancy, cell therapy [autologous (ASCT) or allogeneic hematopoietic stem cell transplantation (allo-SCT), chimeric antigen receptor (CAR) T-cell therapy (CAR-T) or no SCT], cell therapy-related data (i.e. Allo-SCT donor, CAR-T type), last therapy for malignancy (i.e. anti-CD20 monoclonal antibodies) and time from last therapy to SARS-CoV-2 infection, number of SARS-CoV-2 vaccine doses received, Omicron SARS-CoV-2 infection clinical presentation (i.e. symptoms at onset), testing method, laboratory data at diagnosis, and SARS-CoV-2 infection management, severity and outcome (i.e. pneumonia, hospital and/or ICU admission, death). Corticosteroids therapy at the time of SARS-CoV-2 detection was considered when corticosteroids was started before the diagnosis of COVID-19 whereas gamma globulin levels before SARS-CoV-2 detection have not been captured.

### 2.2 Endpoints

The primary endpoint was the description of current strategies to manage COVID-19 in HD patients in the Omicron era among different HDs/procedures and comparison of the outcomes according to whether patients had received cell therapy (ASCT, Allo-SCT, CAR-T). Secondary endpoints included the identification of potential risk factors for different outcomes of interest concerning the severity and outcome of SARS-CoV-2 infection (i.e., COVID-19-related hospital admission, pneumonia development, death, and long-term/prolonged viral shedding).

### 2.3 Definitions

Although we did not sequence SARS-CoV-2 strains in any case, the inference of Omicron VOC was based on the Spanish sequencing epidemiological data (**Supplementary Figure 1**) which started from December 27, 2021, and until July 2023 (21).

Complete vaccination (full primary immunization) schedules were defined as three doses for full primary immunization (and two for the Janssen^®^ COVID-19 Vaccine) according to the current ECIL-9 recommendations (22, 23). An additional dose after completion of full immunization was considered a booster dose.

Breakthrough SARS-CoV-2 infection was defined as molecular (PCR test) or antigenic evidence (antigen test) of SARS-CoV-2 infection in fully vaccinated patients. Most patients underwent weekly PCR test monitoring until negativity of SARS-CoV-2 infection, especially in patients under active treatment for their baseline hematologic disease or in those under active immunosuppression therapy when antiviral therapy was given, or for epidemiological reasons.

Prolonged shedding was defined as persistent PCR positivity after 25 days from the first detection.

Duration of COVID-19 was defined as persistent PCR positivity after 25 days from first detection based on the median time of SARS-Cov-2 detection in our cohort.

Co-infection was defined as a significant co-pathogen detected in concurrent nasopharyngeal or other body site (including urine, blood, or stools) during SARS-CoV-2 infection and until its clinical and/or microbiological resolution.

### 2.4 Statistical analysis

Patient and disease characteristics were reported by descriptive statistics on the total available information. Medians and ranges were used to report continuous variables and counts, and percentages were used for categorical variables. Comparisons between categorical variables were performed using the Chi-squared or the Fisher’s exact test, and continuous variables were compared using the Mann-Whitney U test or the Kruskal-Wallis test when appropriate.

Univariate and multivariate analyses of clinical, laboratory and therapeutic variables associated with COVID-19-related hospital admission, pneumonia development, COVID-19 mortality, and long-term viral shedding were calculated using logistic regression models. For multivariate analysis, only variables with parameter estimates showing a p-value ≤0.1 in the univariate analysis were ultimately included.

A two-sided p-value <0.05 was considered statistically significant. Analyses were performed using the statistical software SPSS v. 25 (IBM SPSS Statistics, Armonk, New York, USA).

## 3 Results

### 3.1 Patient characteristics

**Figure 1:**
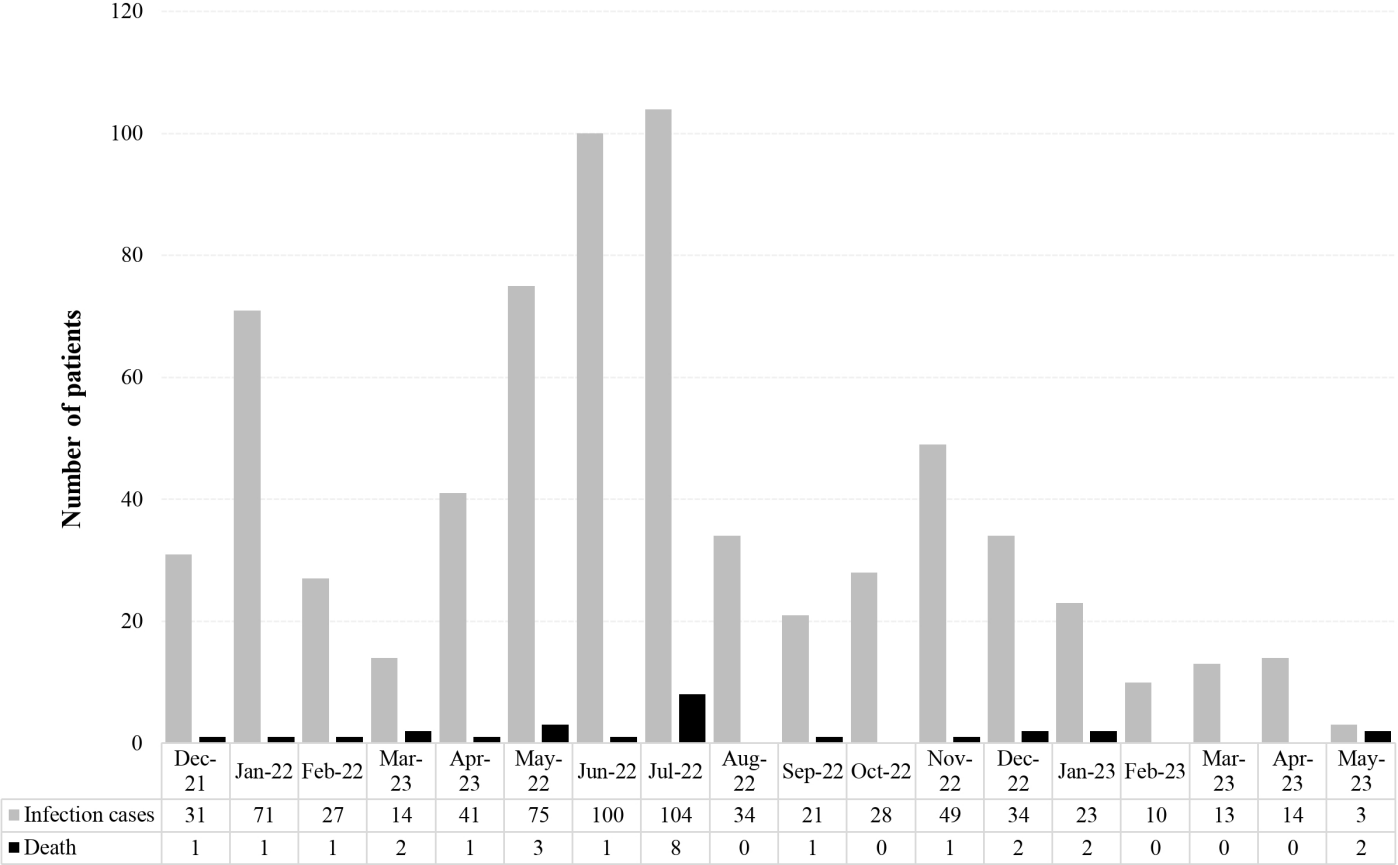
Epidemiological distribution of reported SARS-CoV-2 breakthrough infections and related deaths in the whole cohort.

Overall, the study included 692 patients with HD and confirmed SARS-CoV-2 infection in the Omicron era. **Figure 1** shows the distribution of infection cases during the study period. The majority (n=421; 60.8%) had not received cell therapy (non-SCT patients). Among patients undergoing cell therapy, 238 (34.4%) had received SCT (allogenic: n=156, 22.5%; autologous: n=82, 11.8%) or CAR-T therapy (n=33). The demographic and clinical characteristics of patients according to whether or not they received SCT (ASCT or Allo-SCT) or CAR-T are shown in **Table 1**. Non-SCT patients were of older age (p<0.0001), with 50% of patients aged >71 years at the time of SARS-CoV-2 infection. We found a significantly different distribution of the malignancy between groups (p <0.0001). The most prevalent hematologic disease was B-cell non-Hodgkin’s lymphoma (NHL) for those who did not receive transplant or CAR-T, plasma cell disorders for ASCT recipients and acute myeloid leukemia (AML) for Allo-SCT. Of note, most of the patients had previously received at least 3 vaccine doses for SARS-CoV-2 infection (fully vaccinated), with 82% and 76% of patients who had undergone an ASCT and Allo-SCT, respectively, and 77% of non-SCT patients received ≥3 vaccine doses. Overall, 57% of patients receiving CAR-T were given at least a third vaccine dose (p=0.5). Almost three quarters of patients who did not receive SCT (74%) had received the last therapy for their HD management less than 6 months before the SARS-CoV-2 infection compared with nearly half of patients receiving CAR-T and 30% of recipients of Allo-SCT (p <0.0001). Specifically, Anti-CD20 monoclonal antibody therapy was given to 205 patients (29%), and for most of them (n= 147, 89%), this was in the 6 months before the SARS-CoV-2 infection. A higher percentage of non-SCT patients presented comorbidities such as arterial hypertension (p<0.01) or cardiomyopathy (p=0.021) compared with patients undergoing CAR-T cell therapy or SCT.

**Table 1:** Demographic, clinical, and laboratory characteristics of patients with breakthrough SARS-CoV-2 infection according to cell therapy.

Characteristics	Hematological disease, non-SCT (n = 421)	CAR-T (n= 33)	ASCT (n= 82)	Allo-SCT (n= 156)	*P* value
**Age (years), median (range)^(1)^**	70 (20–93)	58 (30–79)	59 (19–78)	54 (19–79)	<0.0001
0–40 years, n (%)	30 (7)	1 (3)	7 (9)	35 (22)	<0.0001
41–60 years, n (%)	96 (23)	19 (58)	38 (46)	74 (47)	
61–70 years, n (%)	84 (20)	9 (37)	29 (35)	35 (23)	
>71 years, n (%)	211 (50)	4 (12)	8 (10)	12 (8)	
**Male**, *n (%)*	234 (56)	19 (58)	56 (68)	90 (60)	0.5
Pulmonary/cardiovascular RF, n (%)					
Active smoking	33 (8)	4 (12)	7 (8)	11 (7)	0.5
Arterial hypertension	186 (44)	5 (15)	19 (23)	37 (24)	<0.01
Cardiomyopathy^(2)^	89 (21)	3 (9)	13 (16)	9 (6)	0.021
Pulmonary disease	34 (8)	1 (3)	11 (13)	16 (10)	0.06
**Hematological malignancy at COVID-19 diagnosis, n (%)**					<0.0001
AML	41 (10)	0	8 (10)	50 (32)	
ALL	4 (1)	2 (3)	0	25 (16)	
MDS	29 (7)	0	0	22 (14)	
CMPD	17 (4)	0	0	12 (8)	
B-cell NHL	167 (40)	23 (70)	18 (22)	7 (5)	
T-cell NHL	5 (1)	0	2 (3)	9 (6)	
CLL	54 (13)	1 (3)	0	4 (3)	
Plasmatic cell disorder	79 (19)	7 (21)	46 (56)	5 (3)	
HL	17 (4)	0	8 (10)	12 (8)	
AA or others	10 (3)	0	0	10 (6)	
**Time from last therapy for HD to COVID-19 diagnosis**					<0.0001
<6 months	312 (74)	16 (49)	54 (66)	46 (30)	
6–12 months	40 (10)	4 (12)	4 (5)	12 (8)	
>12 months	69 (16)	13 (39)	24 (29)	98 (63)	
**Time from anti-CD20 therapy to COVID-19 diagnosis, n (%)^(3)^**	165 (39)	12 (36)	21 (26)	7 (4.5)	<0.0001
<6 months	128 (78)	4 (34)	14 (67)	1 (14)	
6–12 months	12 (7)	2 (16)	1 (4)	0	
>12 months or not treated	25 (15)	6 (50)	6 (29)	5 (71)	
Allo-SCT donor, n (%)					
HLA identical sibling				57 (37)	
Unrelated Donor				55 (35)	
Haplo-identical family donor				29 (19)	
UCBT				2 (1)	
Missing				13 (8)	
CAR-T type					
Axicel		13 (39)			
Tisacel		2 (6)			
Anti-BCMA		7 (21)			
ARI-001 (anti-CD19) *		7 (21)			
others		4 (12)			
Number of vaccine doses					
Median (IQR)	3 (0–5)	3 (0–5)	4 (0–6)	3 (0–7)	0.5
N (%)					
0	18 (4)	3 (9)	4 (5)	13 (8)	
1	16 (4)	2 (6)	0	1 (1)	
2	66 (16)	9 (27)	11 (13)	23 (15)	
3	163 (39)	9 (27)	26 (32)	42 (27)	
>3	158 (38)	10 (30)	41 (50)	77 (49)	

AA, aplastic anemia; ALL, acute lymphoblastic leukemia; AML, acute myeloid leukemia; allo-SCT, allogeneic hematopoietic stem cell transplantation; ASCT, autologous hematopoietic stem cell transplantation; BCMA, B-cell maturation antigen; CAR-T, chimeric antigen receptor T-cell therapy; CLL, chronic lymphocytic leukemia; CMPD, chronic myeloproliferative disease; F/U, follow-up; HD, hematological disease; HL, Hodgkin lymphoma; HLA, human leukocyte antigen. NHL, non-Hodgkin lymphoma; MDS, myelodysplastic syndrome; SCT, stem cell transplantation or cell therapy; RF, risk factors; UCBT, Unrelated Umbilical Cord Blood Transplantation.

*Academic CAR-T manufactured by the Hospital Clinic i Provincial de Barcelona, Spain.

^(1)^At the time of SARS-CoV-2 infection.

^(2)^Cardiomyopathy was defined by the patient’s medical history or when the left ventricular ejection fraction (LVEF) ≤ 40%.

^(3)^As last therapy for HD.

### 3.2 Characteristics of SARS-CoV-2 infection in treatment and severity according to whether patients received cell therapy

**Table 2** displays the details of the characteristics of SARS-CoV-2 infection, treatments, and outcome. Most patients had experienced COVID-19 symptoms regardless of whether they had received a SCT or CAR-T, with about 2% of asymptomatic cases among recipients of ASCT and non-SCT patients, and 4% in patients receiving Allo-SCT (p=0.3). The percentage of patients with fever was significantly lower in the Allo-SCT group (p=0.04). There were no significant differences amongst groups concerning occurrence of respiratory symptoms (p=0.5). Overall, 27% of patients receiving CAR-T and 21% of patients undergoing ASCT and non-SCT patients, respectively, developed pneumonia compared with only 8% of patients receiving Allo-SCT.

**Table 2:** Breakthrough SARS-CoV-2 infection characteristics, treatment, severity, and outcome according to cell therapy.

Characteristics	Hematological disease, non-SCT (n = 421)	CAR-T (n= 33)	ASCT (n= 82)	Allo-SCT (n= 156)	*P* value
Diagnostic test, n (%)					
PCR	297 (71)	23 (70)	48 (59)	114 (73)	
Antigen-test based	124 (29)	10 (30)	34 (41)	42 (27)	
**Asymptomatic, n (%)**	8 (2)	0	2 (2.4)	6 (4)	0.3
**Fever, n (%)**	149 (35)	15 (45)	33 (40)	34 (22)	0.04
**Respiratory symptoms, n (%)**	217 (52)	19 (57)	38 (46)	65 (42)	0.5
**Pneumonia, n (%)**	88 (21)	9 (27)	17 (21)	12 (8)	0.02
**COVID-19-related hospital admission, n (%)**	100 (24)	10 (30)	12 (15)	25 (16)	0.2
**ICU admission, n (%)**	10 (2.4)	1 (3)	1 (7)	2 (1)	0.6
**Oxygen support, n (%)**	60 (14)	6 (18)	13 (16)	5 (3)	0.03
**Antiviral COVID-19 therapy, n (%)**					<0.01
None	124 (29)	9 (27)	13 (16)	68 (44)	
Remdesivir	149 (35)	10 (30)	25 (30)	59 (38)	
Nirmatrelvir and Ritonavir	142 (34)	13 (40)	43 (52)	25 (16)	
Remdesivir and Nirmatrelvir and Ritonavir	3 (1)	1 (3)	0	0	
Molnupiravir	3 (7)	0	1 (1)	4 (3)	
Sotrovimab mAb	50 (12)	5 (15)	9 (11)	14 (9)	
**Convalescent plasma, n (%)**	27 (6)	1 (3)	4 (4.9)	8 (5)	0.6
**Corticosteroids as COVID-19 therapy, n (%)**	76 (18)	6 (18)	13 (16)	7 (4.5)	0.02
**Corticosteroids at the time of COVID-19 infection, n (%)**	104 (25)	5 (15)	16 (20)	23 (15)	0.2
Laboratory characteristics at the time of COVID-19 diagnosis					
ANC <0.5× 10^9^/L, n/evaluable (%)	22/186 (12)	1/16 (6)	4/30 (13)	5/73 (7)	0.8
ALC <0.5× 10^9^/L, n/evaluable (%)	59/186 (32)	8/16 (50)	8/30 (26)	15/73 (20)	0.2
CRP >8 m g/dL, n/evaluable, (%)	101/178 (57)	9/16 (56)	14/28 (50)	23/71 (32)	0.08
CT value available, n (%)	192	16	30	46	
CT value, median (range)	20 (5–40)	20.5 (11–40)	20.5 (14–37)	20 (11–40)	0.8
**Clinical recovery from COVID-19, n/evaluable (%)**	313/364 (86)	21/27 (78)	62/71(87)	129/141 (91)	0.3
**PCR monitoring, n/evaluable, %^(1)^**	190 (45)	18 (54)	41 (50)	77 (50)	
**Median time from diagnosis to negativity, days (range)**	25 (2–326)	43 (5–103)	27 (3–237)	29 (3–209)	0.13
**COVID-19 related death, n (%)**	21 (5)	2 (6)	3 (4)	0	0.037
**Median time from COVID-19 diagnosis to COVID-19 related death, days (range)**	19 (4–111)	50 (30–70)	13 (11–58)	–	ns
**Median time from COVID-19 diagnosis to all-cause mortality, days (range)**	46 (4–406)	112 (30–402)	58 (11–412)	150 (9–399)	0.2
**All-cause mortality, n (%)**	55 (12)	6 (18)	7 (8.5)	14 (9)	0.079

ALC, absolute lymphocyte count; allo-SCT, allogeneic hematopoietic stem cell transplantation; ANC, absolute neutrophil count; ASCT, autologous hematopoietic stem cell transplantation; CAR-T, chimeric antigen receptor T-cell therapy; CRP, C-reactive protein; CT, PCR cycle threshold; ICU, intensive care unit; mAb, monoclonal antibody; PCR, polymerase chain reaction; SCT, stem cell transplantation or cell therapy. ^(1)^Performed weekly until negative for SARS-CoV-2.

Regarding laboratory data at the time of SARS-CoV-2 infection, the percentage of patients with severe neutropenia (p=0.8) and lymphopenia (p=0.2) were also comparable between the groups.

Moreover, the percentage of patients requiring hospitalization due to COVID-19 did not significantly differ between groups (p=0.2). Only two patients receiving Allo-SCT and one patient of those treated with CAR-T and ASCT, respectively, required ICU admission (p=0.6). Oxygen support was required by 18%, 16% and 3% of patients receiving CAR-T, ASCT and Allo-SCT, respectively, and by 14% of non-SCT patients (p=0.03).

Management with or without antiviral therapy for SARS-CoV-2 infection significantly differed between groups with higher rate of untreated patients (44%) in the allo-SCT group (p <0.01). Overall, 214 out of 692 (31%) did not receive any of the authorized COVID-19 therapies. Among patients who received antiviral therapy, most patients received remdesivir (30% of patients receiving CAR-T and ASCT respectively, with 38% receiving Allo-SCT, and 35% of non-SCT patients) or nirmatrelvir plus ritonavir (40%, 52% and 16% of patients receiving CAR-T, ASCT and Allo-SCT, respectively, and 34% of non-SCT patients).

The use of corticosteroids for SARS-CoV-2 infection management was also significantly different between groups (p=0.02), with 18% of non-SCT patients and of those patients receiving CAR-T, respectively, and 16% of ASCT patients being treated with corticosteroids (both with and without a concomitant antiviral) compared to 4.5% of patients receiving Allo-SCT.

### 3.3 SARS-CoV-2 infection outcome according to whether patients received cell therapy

Data in **Table 2** shows that at the time of the last follow-up, there was a high percentage of patients with full clinical recovery from COVID-19, with 91% and 87% of Allo-SCT and ASCT recipients, respectively, and 86% of non-SCT patients, achieving full recovery (p= 0.3). There were no significant differences in time to SARS-CoV-2 PCR negative result between groups (p=0.13).

Eighty-two patients died (11.8%) in the whole cohort at a median of 59 days (range 4–412) after the detection of SARS-CoV-2. Causes of death included progression or relapse of the underlying HD (n=35, 43%), transplant-related complications (n= 9, 11%) and others (n= 12, 15%). Twenty-six patients’ (3.7%) death was directly attributable to COVID-19, whereas COVID-19-related mortality in hospitalized patients was 14%. There were significant differences between the groups in the rate of COVID-19-related death, occurring in 6% and 4% of patients receiving CAR-T and ASCT, respectively, and in 5% of non-SCT patients, while none of the patients who had received an allo-SCT had died (p=0.037). The all-cause mortality rate was higher in patients who had received CAR-T (18%) as compared with ASCT (8.5%) and allo-SCT (9%) and non-SCT patients (12%), without reaching statistical significance.

### 3.4 Characteristics, severity, and outcome of breakthrough SARS-CoV-2 infection according to whether antiviral therapy was used or not

A total of 478 out of 692 (69%) patients were treated with antiviral drugs. Among the treated patients were 243 (50%) who received remdesivir, 223 (47%) nirmatrelvir/ritonavir, 4 (1%) received a combination of both and 8 (2%) molnupiravir. Characteristics, severity, and outcome of breakthrough SARS-CoV-2 infections were compared according to whether patients had received antiviral therapy (AVT) with remdesivir or nirmatrelvir/ritonavir, respectively, or who had not received any AVT as specific therapy for COVID-19 (**Table 3**).

**Table 3:** Patients’ characteristics and breakthrough SARS-CoV-2 infection characteristics, treatment, severity and outcome according to antiviral therapy.

Characteristics	No ATV (n = 214)	Remdesivir (n=243)	Nirmatrelvir/ritonavir (n= 223)	*P* value
**Male sex**, n (%)	113 (53)	150 (62)	131 (59)	0.3
**Age (years), n (%)^(1)^**				0.12
< 41 years	35 (16)	18 (7)	19 (9)	
41–60 years	69 (32)	86 (35)	66 (30)	
61–70 years	46 (22)	49 (21)	60 (26)	
>71 years	64 (30)	90 (37)	79 (35)	
Pulmonary/cardiovascular risk factors, n (%)				
Active smoking	12 (6)	21 (9)	21 (9)	0.6
Arterial hypertension	61 (29)	105 (43)	79 (35)	0.045
Cardiomyopathy^(2)^	32 (15)	49 (20)	30 (13)	0.6
Pulmonary disease	14 (7)	33 (14)	15 (7)	0.2
**Diagnostic testing for COVID-19, n (%)**				0.001
PCR	120 (56)	218 (90)	135 (61)	
Antigen-test based	94 (44)	25 (10)	88 (39)	
**Time from last therapy for the HD to COVID-19 diagnosis, n (%)**				<0.001
<6 months	80 (37)	182 (75)	158 (71)	
6–12 months	21 (10)	19 (8)	20 (9)	
>12 months or not treated	113 (53)	42 (17)	45 (20)	
**Prior anti-CD20 therapy for the HD, n (%)**	38 (18)	74 (30)	88 (39)	<0.001
**Time from anti-CD 20 therapy to COVID-19 diagnosis, n (%)**				<0.001
No anti-CD20	176 (82)	168 (69)	135 (61)	
<6 months	19 (9)	57 (23)	68 (30)	
6–12 months	6 (3)	6 (2)	3 (1)	
>12 months or not treated	13 (7)	12 (5)	17 (8)	
**Prior vaccine doses, n (%)**				<0.001
0	2 (1)	22 (10)	14 (6)	
1	2 (1)	8 (3)	8 (4)	
2	21 (10)	56 (23)	29 (13)	
≥3	189 (88)	157 (64)	172 (77)	
**Asymptomatic, n (%)**	0	7 (3)	1 (0.5)	0.04
**Fever, n (%)**	31 (14)	127 (52)	71 (32)	<0.001
**Respiratory symptoms, n (%)**	56 (26)	172 (71)	107 (48)	<0.001
**Pneumonia, n (%)**	15 (8)	97 (40)	14 (6)	<0.001
**COVID-19-related hospital admission, n (%)**	10 (5)	118 (48)	19 (8)	<0.001
**ICU admission, n (%)**	2 (1)	14 (6)	1 (0.5)	0.002
**Oxygen support, n (%)**	10 (5)	69 (28)	5 (2)	<0.001
**Co-infections, n (%)**	19 (9)	46 (19)	9 (5)	<0.001
**Sotrovimab mAb, n (%)**	23 (11)	47 (19)	7 (4)	<0.001
**Convalescent plasma, n (%)**	4 (2)	30 (12)	6 (3)	<0.001
**Corticosteroids as COVID-19 therapy, n (%)**	6 (3)	79 (33)	6 (3)	<0.001
**Corticosteroids at the time of COVID-19 infection, n (%)**	29 (14)	68 (28)	48 (22)	0.002
Laboratory data at the time of SARS-CoV-2 detection				
ANC <0.5× 10^9^/L, n (%)	4 (2)	18 (7)	10 (5)	0.69
ALC <0.5× 10^9^/L, n (%)	10 (5)	50 (21)	30 (13)	0.06
CRP >8 mg/dL, n (%)	18 (8)	76 (31)	50 (22)	0.028
**CT values at diagnosis, median (range)**	19 (2–37)	20 (2–40)	21 (9–40)	0.3
**Recovery from COVID-19 (n/evaluable, %)**	181/184 (98)	163/189 (86)	170/173 (98)	0.001
**Median time from diagnosis to negativity, days (range)**	24.5 (3–236)	31 (2–209)	22 (2–327)	0.005
**Prolonged shedding, n/evaluable (%)**	30/62 (48)	80/128 (63)	122 (40)	0.002
**COVID-19 related death, n (%)**	2 (0.9)	21 (8.6)	3 (1)	<0.001
**Median time from diagnosis to death (range)**	11.5 (10–13)	30 (4–111)	24 (18–77)	0.04

ANC, absolute neutrophil count; ALC, absolute lymphocyte count; ATV, antiviral therapy, CT, PCR cycle threshold; CRP, C-reactive protein; mAb, monoclonal antibody.

^(1)^At study inclusion.

^(2)^Cardiomyopathy was defined by the patient’s medical history or when the left ventricular ejection fraction (LVEF) ≤ 40%.

Overall, 75% and 71% of patients receiving AVT with remdesivir and/or nirmatrelvir/ritonavir had received the last therapy for hematologic malignancy in the 6 months before the SARS-CoV-2 infection compared with 37% of patients who had not received AVT, in whom this period was mainly more than 12 months (p<0.001). Treatment with prior anti-CD20 mAbs and time from anti-CD20 therapy completion to SARS-CoV-2 infection was also significantly different between groups (p<0.001). A higher percentage of patients receiving remdesivir had arterial hypertension (43%) compared with those treated with nirmatrelvir/ritonavir (35%) or who did not receive AVT (29%) (p=0.045). Overall, 88% of non-AVT patients had received more than 3 vaccine doses compared to 77% and 64% of patients receiving remdesivir and nirmatrelvir/ritonavir, respectively (p<0.001). A higher percentage of patients treated with remdesivir had respiratory symptoms (71%) when compared with patients treated with nirmatrelvir/ritonavir (48%) and patients that did not receive AVT (26%) for COVID-19 management (p<0.001). A higher percentage of patients receiving remdesivir had pneumonia (40%), required COVID-19-related hospitalization (48%) and ICU admission (6%), and needed oxygen support (28%) compared to patients treated with nirmatrelvir/ritonavir and patients who had not received AVT (all p<0.001 between groups). Other authorized treatments for COVID-19 also differed according to whether or not they received AVT. Thus, a higher percentage of patients treated with remdesivir received corticosteroids (33% vs. 3%) and convalescent plasma (12% vs. 3% and 2%) when compared to nirmatrelvir/ritonavir and no AVT, respectively (all p<0.001). COVID-19-related mortality was significantly lower in patients who had not received AVT (0.9%) and in patients receiving nirmatrelvir/ritonavir (1%) compared with those treated with remdesivir (8.6%).

### 3.5 Risk factors associated with hospital admission and pneumonia due to SARS-CoV-2 infection

**Figure 2:**
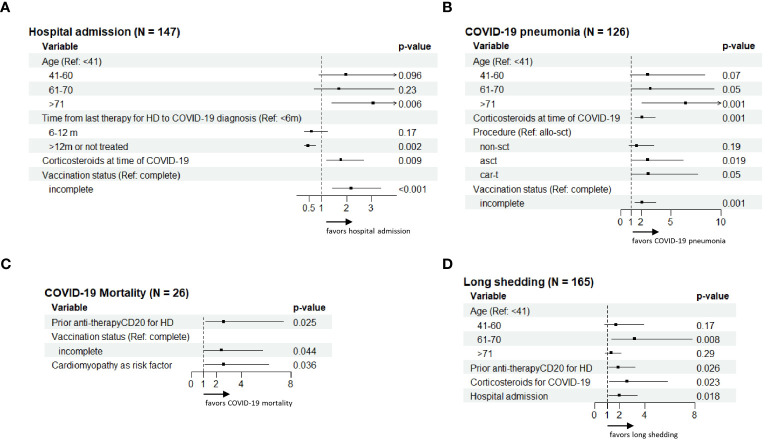
Exploratory forest plot analysis for the risk factors concerning hospital admission **(A)**, COVID-19 pneumonia **(B)**, COVID-19 mortality **(C)** and long-term viral shedding **(D)** shown in multivariate analysis in **Tables 4**, **5**.

The hospital admission rate in the current series was 23% (159 out of 692), and 126 (18%) developed pneumonia. Data on logistic regression univariate and multivariate analyses of factors associated with SARS-CoV-2 infection-related hospital admission and pneumonia are shown in **Table 4**, with the most relevant represented as forest plot in **Figure 2**. Multivariate analysis revealed that independent factors significantly associated with hospital admission were: age >70 years (OR 3.08, 95% CI 1.4–6.9, p=0.006), treatment with corticosteroids at the time of COVID-19 diagnosis (OR 1.78, 95% CI 1.2–2.7, p=0.009), a time period >12 months since last therapy for the HD before SARS-CoV-2 infection (OR 0.46, 95% CI 0.3–075, p=0.002), and incomplete vaccination status (OR 2.18, 95% CI 1.42–3.36, p<0.001).

**Table 4:** Univariate and multivariate analysis of risk factors for COVID-19 related hospital admission and pneumonia.

Variables	Hospital admission* (n=147)				COVID-19 Pneumonia (n=126)			
	Univariate analysis		Multivariate analysis		Univariate analysis		Multivariate analysis	
	OR (95% C.I.)	*P* value	OR (95% C.I.)	*P* value	OR (95% C.I.)	*P* value	OR (95% C.I.)	*P* value
Age (years)^(1)^								
< 41 years	1		1		1		1	
41–60 years	1.85 (0.86–4)	0.114	1.97 (0.89–4.4)	0.096	3.1 (1.08–9.1)	0.036	2.7 (0.91–8.4)	0.07
61–70 years	1.47 (0.65–3.3)	0.34	1.7 (0.7–3.9)	0.23	3.27 (1.1–9.8)	0.034	2.96 (1–9.3)	0.05
>71 years	2.66 (1.25–5.7)	0.011	3.08 (1.4–6.9)	0.006	6.2 (2.2–17.6)	0.001	6.5 (2.1–20)	0.001
**Sex (male/female)**	1.29 (0.88–1.88)	0.17	ns		1.29 (0.8–1.9)	0.2		
**Pulmonary/cardiovascular risk factors, n (%)**							ns	
Active smoking	0.8 (0.39–1.65)	0.56			1.13 (0.59–2.26)	0.72		
Arterial hypertension	1.43 (0.98–2.1)	0.057			2.05 (1.4–3.0)	<0.001		
Cardiomyopathy^(2)^	1.55 (0.98–2.46)	0.058			1.75 (1.08–2.8)	0.021		
Pulmonary disease	2 (0.42–8.8)	0.35			2.5 (0.67–9.8)	0.2		
**Hematological malignancy**							ns	
AML	1				1			
ALL	–				–			
MDS	0.56 (0.17–1.8)	0.35			0.97 (0.41–2.3)	0.9		
CMPD	0.58 (0.14–2.4)	0.41			1.04 (0.37–2.9)	0.9		
B-cell NHL	1.13 (0.52–2.4)	0.75			1.7 (0.95–3.0)	0.07		
T-cell NHL	–				–			
CLL	0.78 (0.26–1.8)	0.41			1.13 (0.51–2.48)	0.76		
Plasmatic cell disorder	0.69 (0.26–1.8)	0.46			0.7 (0.36–1.4)	0.34		
HL	0.58 (0.14–2.4)	0.46			0.6 (0.21–1.8)	0.38		
AA or others	2.7 (0.15–47)	0.49			1.33 (0.25–2.9)	0.9		
Time from last therapy for HD to COVID-19 diagnosis								
<6 months	1		1		1			
6–12 months	0.55 (0.27–1.1)	0.1	0.6 (0.3–1.25)	0.17	0.68 (0.32–1.4)	0.68		
>12 months or not treated	0.3 (0.21–056)	<0.001	0.46 (0.3–0.75)	0.002	0.64 (0.4–1)	0.056		
**Prior anti-CD20 therapy for the HD**	1.46 (0.99–2.14)	0.055			1.67 (1.1–2.5)	0.012		
Time from anti-CD 20 therapy to COVID-19^(3)^								
No anti-CD20	1				1			
<6 months	1.67 (1.1–2.5)	0.01			1.56 (0.99–2.46)	0.057		
6–12 months	0.64 (0.14–2.9)	0.5			2.69 (0.89–8.1)	0.077		
>12 months or not treated	1.07 (0.49–2.3)	0.8			2.02 (0.99–4.1)	0.051		
**Corticosteroids at the time of COVID-19**	1.96 (1.3–2.95)	0.001	1.78 (1.2–2.7)	0.009	2.1 (1.34–3.2)	0.001	2.1 (1.4–3.4)	0.001
**Procedure**							ns	
Non-SCT	1.6 (1.01–2.64)	0.04			3.1 (1.68–5.9)	<0.001	1.6 (0.78–3.3)	0.19
Allo-SCT	1				1		1	
ASCT	0.89 (0.42–1.9)	0.77			3.1 (1.42–6.9)	0.005	2.7 (1.17–6.2)	0.019
CAR-T	2.27 (0.96–5.3)	0.06			4.5 (1.7–11.8)	0.002	2.77 (1.0–7.7)	0.05
**Vaccination status†**				<0.001				0.001
Complete	1		1		1		1	
Incomplete	1.99 (1.33–2.95)	0.001	2.18 (1.42–3.36)		1.55 (1.01–2.37)	0.044	2.1 (1.35–3.48)	
Co-infection**	2.45 (1.4–4.1)	0.001	NT		10.8 (6.4–18.4)	<0.001	NT	
Laboratory data at diagnosis of breakthrough SARS-CoV-2 infections								
CT value*			NT					
≥25	1				1			
<25	2.24 (1.23–4.0)	0.008			0.75 (0.4–1.3)	0.7		
CRP >8 mg/dL*	1.7 (1.1–2.7)	0.023	NT		4.78 (2.5–8.9)	<0.001	NT	
ALC < 0.5 × 109/L*	1.4 (0.87–2.35)	0.14	NT		1.43 (0.87–2.3)	0.14	NT	
ANC < 0.5 × 109/L*	0.95 (0.46–1.99)	0.9	NT		1.45 (0.63–3.3)	0.37	NT	

AA, aplastic anemia; ALC, absolute lymphocyte count; ALL, acute lymphoblastic leukemia; allo-SCT, allogeneic hematopoietic stem cell transplantation; AML, acute myeloid leukemia; ANC, absolute neutrophil count; ASCT, autologous hematopoietic stem cell transplantation; CLL, chronic lymphocytic leukemia; CMPD, chronic myeloproliferative disease; CT, PCR cycle threshold; CRP, C-reactive protein; HD, Hodgkin lymphoma; MDS, myelodysplastic syndrome; NHL, non-Hodgkin lymphoma; ns, not significant; NT, not tested; SCT, stem cell transplantation.

^(1)^At study inclusion.

^(2)^Cardiomyopathy was defined by the patient’s medical history or when the left ventricular ejection fraction (LVEF) ≤ 40%.

^(3)^As last therapy for HD.

*These variables have not been tested since more than there is a lack of data in more than 40% of cases.

**This variable was not included in the multivariate analyses due to the collinearity with hospital admission and pneumonia.

†Complete vaccination was defined as at least 3 vaccine doses, whereas incomplete include 2 or less.

Factors independently associated with the development of COVID-19 pneumonia on multivariate analysis were also older age (>70 years: OR 6.5, 95% CI 2.1–20, p=0.001), treatment with ASCT (OR 2.7, 95% CI 1.17–6.2, p=0.019) and CAR-T cell therapy (OR 2.77, 95% CI 1.0–7.7, p=0.05), use of corticosteroids at the time of SARS-CoV-2 infection (OR 2.1, 95% CI 1.4–3.4, p=0.001), and incomplete vaccination status (OR 2.10, 95% CI 1.35–3.48, p=0.001).

### 3.6 Risk factors associated with COVID-19 mortality and long-term SARS-CoV-2 shedding

We also assessed the risk factors associated with breakthrough SARS-CoV-2 infection mortality and long-term viral shedding of SARS-CoV-2 using logistic regression univariate and multivariate analyses (**Table 5**, **Figure 2**).

**Table 5:** Univariate and multivariate analysis of risk factors for COVID-19 mortality and long-term viral shedding.

Variables	COVID-19 mortality* (n=26)				Long-term viral shedding (>25 days from diagnosis) (n= 165 out of 319)			
	Univariate analysis		Multivariate analysis		Univariate analysis		Multivariate analysis	
	OR (95% C.I.)	*P* value	OR (95% C.I.)	*P* value	OR (95% C.I.)	*P* value	OR (95% C.I.)	*P* value
**Age (years)^(1)^**			ns					
< 41 years	1				1		1	
41–60 years	0.96 (0.09–9.4)	0.9			2.04 (0.92–4.5)	0.079	1.7 (0.77–3.9)	0.17
61–70 years	2.8 (0.33–24)	0.33			3.77 (1.6–8.9)	0.003	3.2 (1.35–7.8)	0.008
>71 years	5.2 (0.7–40)	0.1			2.66 (1.16–6)	0.02	1.3 (0.8–2.1)	0.29
**Sex (male/female)**	0.59 (0.27–1.3)	0.2			1.01 (0.64–1.58)	0.95	2.19 (0.94–5.07)	0.067
Hematological malignancy								
AML	1		ns		1		ns	
ALL	–				–			
MDS	1.1 (0.3–4.3)	0.9			0.21 (0.21–1.4)	0.1		
CMPD	–				–			
B-cell NHL	1.67 (0.5–5.2)	0.37			0.61 (0.22–1.65)	0.34		
T-cell NHL	1.6 (0.16–15.3)	0.7			0.26 (0.02–3.52)	0.31		
CLL	2.2 (0.57–8.6)	0.2			0.74 (0.2–2.7)	0.64		
Plasmatic cell disorder	0.17 (0.01–1.6)	0.12			0.41 (0.13–1.3)	0.13		
HL	0.66 (0.07–6.1)	0.7			0.06 (0.01–0.57)	0.015		
AA or others	–				–			
**Time from last therapy HD to COVID-19 diagnosis**			ns					
<6 months	1				1			
6–12 months	1.6 (0.51–4.9)	0.4			0.59 (0.27–1.25)	0.17		
>12 months or not treated	0.45 (0.15–1.3)	0.16			0.89 (0.51–1.56)	0.69		
**Prior anti-CD20 therapy for the HD**	2.9 (1.3–6.4)	0.008	2.6 (1.12–7.4)	0.025	1.4 (0.9–2.28)	0.12	1.86 (1.1–3.2)	0.026
**Time from anti-CD 20 therapy to COVID-19, n (%)**			ns					
No anti-CD20	2.2 (0.91–5.6)	0.07			1.4 (0.86–2.3)	0.16		
<6 months	9.87 (2.4–39.6)	0.001			1.06 (0.25–4.3)	0.93		
6–12 months	2.8 (0.78–10.6)	0.11			1.7 (0.61–5)	0.28		
>12 months or not treated	1				1			
**Corticosteroids at the time of COVID-19 diagnosis**	1.37 (0.6–3.3)	0.5			0.79 (0.48–1.3)	0.38		
**Procedure**			ns					
Non-SCT	8.1 (1.1–61)	0.04			0.77 (0.45–1.33)	0.35		
Allo-SCT	1				1			
ASCT	5.88 (0.6–57)	0.12			0.91 (0.42–1.98)	0.82		
CAR-T	10 (0.87–113)	0.063			2.15 (0.69–6.7)	0.18		
**Vaccination status†**							ns	
Complete	1		1		1			
Incomplete	2.04 (0.9–4.59)	0.084	2.41 (1.02–5.69)	0.044	1.41 (0.86–2.3)	0.16		
Pulmonary/cardiovascular risk factors, n (%)								
Active smoking	0.45 (0.06–3.4)	0.44			1.39 (0.62–3.1)	0.41		
Arterial hypertension	2.9 (1.3–6.57)	0.009	ns		1.05 (0.66–1.67)	0.82		
Cardiomyopathy	3.5 (1.5–8)	0.003	2.6 (1.06–6.2)	0.036	1.17 (0.17–21)	0.59		
Pulmonary disease	1.6 (0.52–5.8)	0.35			2.6 (0.49–13.6)	0.25		
Antiviral COVID-19 therapy **	NT		NT				ns	
None					1			
Remdesivir					1.7 (0.98–3.2)	0.06		
Nirmatrelvir y ritonavir					0.76 (0.38–1.3)	0.28		
Remdesivir and nirmatrelvir/ritonavir					NT			
Molnupiravir					3.2 (0.3–32)	0.32		
Sotrovimab	2.48 (0.95–6.4)	0.06			2.6 (1.3–5.4)	0.007		
Convalescent plasma**	2.2 (0.6–7.9)	0.2	NT		2.6 (1.14–6.25)	0.023	ns	
Corticosteroids for COVID-19**	19.1 (7.7–46)	<0.001	NT		3.7 (1.7–8.1)	0.001	2.58 (1.13–5.8)	0.023
Pneumonia**	44.1 (13–149)	<0.001	NT		2.1 (1.1–3.9)	0.027	ns	
Hospital admission **	2.67 (1.2–5.8)	0.015	NT		2.4 (1.4–4.1)	0.001	1.96 (1.12–3.4)	0.018
Co-infections **	5.6 (2.4–12.6)	<0.001	NT		1.9 (0.87–4.3)	0.11	ns	
Laboratory data at diagnosis of COVID-19								
CT value at diagnosis*			NT				NT	
≥25	1				1			
<25	0.8 (0.27–2.4)	0.72			2.01 (1.01–4)	0.046	ns	
CRP >8 mg/dL*	10.5 (1.3–83)	0.025	NT		1.7 (0.91–3.2)	0.092	NT	
ALC < 0.5 × 10^9^/L*	0.85 (0.1–6.8)	0.8	NT		1.3 (0.71–2.6)	0.33	NT	
ANC < 0.5 × 10^9^/L*	0.89 (0.23–3.4)	0.8	NT		1.1 (0.41–2.9)	0.84	NT	

AA, aplastic anemia; ALC, absolute lymphocyte count; allo-SCT, allogeneic hematopoietic stem cell transplantation; ALL, acute lymphoblastic leukemia; AML, acute myeloid leukemia; ANC, absolute neutrophil count; ASCT, autologous stem cell transplantation; CLL, chronic lymphocytic leukemia; CMPD, chronic myeloproliferative disease; CRP, C-reactive protein; CT, PCR cycle threshold; HD, Hodgkin lymphoma; MDS, myelodysplastic syndrome; NHL, non-Hodgkin lymphoma; ns, not significant; NT, not tested; SCT, stem cell transplantation.

^(1)^At study inclusion.

*These variables have not been tested since more than there is a lack of data in more than 40% of cases.

**These variables were not included in the multivariate analyses due to the collinearity with COVID-19 severity.

†Complete vaccination was defined as at least 3 vaccine doses, whereas incomplete include 2 or less.

The multivariate regression analysis identified the following variables as independent factors significantly associated with COVID-19-related mortality: prior anti-CD20 therapy (OR 2.6, 95% CI 1.12–7.4, p=0.025), incomplete vaccination status (OR 2.41, 95% CI 1.02–5.69, p=0.044), and comorbid cardiomyopathy (OR 2.6, 95% CI 1.06–6.2, p=0.036).

Patient and disease characteristics identified as independent factors significantly associated with prolonged viral shedding of SARS-CoV-2 by multivariate analysis included prior anti-CD20 therapy (OR 1.86, 95% CI 1.1–3.2, p=0.026), treatment with corticosteroids for breakthrough SARS-CoV-2 infection (OR 2.58, 95% CI 1.13–5.8, p=0.023), and hospital admission (OR 1.96, 95% CI 1.12–3.4, p=0.018).

## 4 Discussion

This real-life study was performed through a national retrospective multicenter registry launched by the GETH-TC in a large cohort of 692 consecutive patients with HD and confirmed SARS-CoV-2 infection during the Omicron period. This study assessed the management strategies used in real-life practice and clinical characteristics of SARS-CoV-2 infection, severity, and outcome according to the HD type and/or cell therapy procedures. AVT was mostly given to patients harboring conditions associated with higher risk of severe COVID-19. Remdesivir was the treatment of choice for moderate-to-severe COVID-19 whereas nirmatrelvir/ritonavir was used for mild-to-moderate COVID-19. Nearly one-third of HD patients did not receive any of the authorized SARS-CoV-2 therapies and had an overall favorable outcome. The current series was able to identify risk factors for adverse clinical outcome in this highly immunocompromised cohort. A notably low mortality rate, as compared to prior SARS-CoV-2 VOC waves, was registered throughout the study. Older age, ASCT, CAR-T therapy, corticosteroids at the time of COVID-19 diagnosis and incomplete vaccination status were factors independently associated with COVID-19 severity that were significantly related with a higher likelihood of hospital admission and pneumonia development. Incomplete vaccination status, treatment with prior anti-CD20 mAbs, and comorbid cardiomyopathy were identified as independent risk factors for COVID-19 mortality, with no impact of underlying hematologic malignancy. Longer SARS-CoV-2 detection was associated with age, anti-CD20 mAbs, the use of corticosteroids and hospitalization.

This retrospective study produced important observations regarding SARS-CoV-2 management in HD patients in a real-world setting. To begin with, 31% of the series did not receive any therapy against SARS-CoV-2 infection but showed a very low COVID-19 related mortality (0.9%). These results suggest that not every HD patient with SARS-CoV-2 infection would run a severe course in the absence of AVT, in particular those without comorbidities, no anti-CD20 therapy in the last 12 months, last HD therapy given more than 12 months before SARS-CoV-2 detection, without corticosteroids, fully vaccinated (3 doses), low inflammatory levels at diagnosis and without fever and/or respiratory symptoms. Antivirals were mostly given to patients harboring fever and respiratory symptoms as well as conditions already identified as risk factors for severe COVID-19, such as recent HD therapy, prior anti-CD20 therapy, corticosteroids at the time of infection or incomplete vaccination (24). Indeed, the updated recommendations for treating COVID-19 in HD patients highlight the use of AVT in every HD patient with mild-to-severe infection, and the present study employed each antiviral agent accordingly except in those at lower risk of severe COVID-19. Thus, nirmatrelvir/ritonavir was predominantly used for at-risk patients with mild infections in outpatient scenarios, since its use demonstrated a lower hospitalization rate or death in symptomatic, non-hospitalized, adult patients with COVID-19 (25). Concerning remdesivir, a systematic review of nine RCTs and the Infectious Diseases Society of America (IDSA) guidelines recommend remdesivir administration during COVID-19 in those patients with mild COVID-19 and no need of oxygen support, showing a reduction in mortality (26–28). Reports for nirmatrelvir/ritonavir and remdesivir as therapies for SARS-CoV-2 infection in healthy adults also match these findings (29–31). In the present study, remdesivir was the preferred option for treating severe cases in hospitalized patients, which could potentially account for the notably elevated COVID-19-related mortality observed in the remdesivir cohort. Specific analyses on factors associated with SARS-CoV-2 outcomes in the treated cohort has been recently published (32). Of note, none of the allo-SCT recipients died due to SARS-CoV-2 infection. The two deaths reported among the untreated were in patients older than 75 years in the terminal phase of their hematologic disease and, therefore, not treated for SARS-CoV-2 infection.

The underlying hematologic malignancy was not significantly associated with a requirement of hospital admission, pneumonia development or COVID-19-related survival. This data aligns with a recent analysis from the EPICOVIDEHA registry that demonstrated no effect of underlying hematologic malignancy on survival in a large cohort of vaccinated patients with HD who had developed breakthrough COVID-19 (33). However, these results might suggest that more important than the disease itself is the disease status along with the type and timing of last therapy. In the later scenarios, particular attention is warranted in those who receive anti-CD20 therapy due to the increased risk of severe disease, prolonged SARS-CoV-2 detection and/or long-term COVID-19-associated comorbidities. In fact, a previous study performed with this registry reported that treatment with prior anti-CD20 mAbs decreases the probability of producing reactive antibodies against SARS-CoV-2 after vaccination (34). The present study has identified prior treatment with anti-CD20 mAbs as an independent factor significantly associated with COVID-19 mortality and prolonged viral shedding by multivariate analysis. Indeed, high COVID-19 mortality was reported in patients with prior anti-CD20 therapy, especially in those infected with the SARS-CoV-2 Omicron variant, and even in fully vaccinated hematologic patients (35, 36). In the context of autoimmune diseases, it is known that B-cell depletion impairs SARS-CoV-2 antibody production (37–40), which is probably the underlying cause for a poor virus response – natural or vaccination-triggered – leading to severe COVID-19 symptoms in immunocompromised patients treated with anti-CD20.

Furthermore, COVID-19-related death was low in our series when compared to prior waves, ranging from 0%, 4% and 6% in Allo-SCT, ASCT and CAR-T recipients and 5% in non-SCT patients, with significant differences between groups in descriptive analyses (41, 42). Previous reports in pooled rates for CAR-T recipients showed up to 40% mortality after SARS-CoV-2 infection (41). The fact that the CAR-T recipients in our analysis presented a lower percentage of comorbidities – as arterial hypertension and cardiomyopathy – when compared to HD patients receiving other procedures may had influenced this low mortality, as well as adequate concurrent antiviral therapy with corticosteroids, as specially recommended (43). A prior analysis of our group reported lower mortality also in recipients of SCT as compared to non-SCT patients in the pre-vaccination era supporting a more favorable outcome in this subset of patients (44). However, although a non-SCT strategy was associated with a higher risk of COVID-19 mortality by univariate analysis, it was not identified as an independent factor associated with mortality due to breakthrough SARS-CoV-2 infections by multivariate analysis in our series. Nevertheless, mortality was still significant (14%) in HD patients requiring hospital admission, which warrants improvement in the management. Regarding risk factors for SARS-CoV-2 infection outcomes, older age has been previously correlated with high mortality after SARS-CoV-2 infection in the general population, and the same occur in HD patients (13, 44–46). Age >70 years was identified as an independent risk factor for COVID-9-related pneumonia and hospital admission although not significantly associated with mortality due to SARS-CoV-2 infection, probably attributable to the low number of patients who died from COVID-19 in our series.

Most patients enrolled in this study received more than three SARS-CoV-2 vaccine doses; a trait previously shown as predictor for COVID-19 treatment success in HD patients (13). Although patients with a weakened immune system respond poorly to vaccines, SARS-CoV-2 booster administrations have been shown to significantly improve the concentration of neutralizing antibodies, and more so in individuals with B-cell depletion (47–49). A third vaccination leads to antibody maturation, and a fourth booster aims for lasting immunity and a humoral response more comparable to that of healthy individuals (11, 48). Multivariate analyses performed in our study identified incomplete vaccination status as an independent factor associated with COVID-19-related mortality in patients with HD with breakthrough SARS-CoV-2 infections. However, complete vaccination schedules included three doses for full primary immunization (two for the Janssen^®^ COVID-19 Vaccine) at the time of study conduct as recently recommended (23) and an additional dose after completion of full immunization was considered as a booster dose. Thus, the potential impact of booster administration on the risk of higher severity and worse outcome following breakthrough SARS-CoV-2 infection was not specifically analyzed in our study. For those HD patients where vaccination may not be a sufficient strategy, the use of monoclonal antibodies in pre-exposure prophylaxis should be considered to minimize the risk of reinfection or severe forms of COVID-19, as indicated by the updated GELLC guidelines (50).

Corticosteroid anti-inflammatory action is important to ameliorate severe inflammatory response against SARS-CoV-2 infections in the healthy population during the first waves, but it may also interfere with antibody production (51, 52). Treatment with corticosteroids during COVID-19 was previously associated with lower neutralizing antibodies levels after vaccination in HD patients (53). The use of corticosteroids at the time of breakthrough SARS-CoV-2 infection detection was a risk factor for COVID-19 hospital admission and pneumonia. This circumstance has also been observed in allo-SCT recipients with several common seasonal respiratory viruses (54). This finding and prior experience with other respiratory viruses raises serious concerns about the potential benefit of corticosteroids in managing moderate-to-severe COVID-19 in these highly immunocompromised patients and highlights the critical need for randomized clinical trials with corticosteroids in said population before assuming the same benefit as in the healthy population.

This study has certain limitations, such as its retrospective nature, the limited amount of data evaluable for laboratory parameters at the time of SARS-CoV-2 detection and the use of several PCR tests with different analytical performance. To our knowledge, this is the first and the most extensive real-world study describing current COVID-19 management strategies and assessing the clinical presentation, severity, and outcome of breakthrough Omicron SARS-CoV-2 infections in HD patients.

In conclusion, this real-world study conducted with a large cohort of patients reports on the current management strategies of SARS-CoV-2 infection in HD patients, which was mainly based on the perception of the severity of risk of each individual and the clinical infection severity. We provide important risk factors for pneumonia, hospital admission, COVID-19 mortality, and long-term viral shedding of breakthrough SARS-CoV-2 infections in the post-vaccination era. It reinforces previous observations that patients with B-cell depletion, older age, cardiomyopathy, and who have received fewer than three vaccine doses should receive antiviral COVID-19 treatment as early as possible together with more thorough disease monitoring. A complete vaccination status (3 doses) should be pursued in healthcare systems and its campaigns in HD patients.

## Data availability statement

The original contributions presented in the study are included in the article/**Supplementary Material**. Further inquiries can be directed to the corresponding author.

## Ethics statement

The local Research Ethics Committee of the Hospital Clínico Universitario de Valencia approved the study (reference code 35.21). The studies were conducted in accordance with the local legislation and institutional requirements. The participants provided their written informed consent to participate in this study.

## Author contributions

JP: Conceptualization, Data curation, Formal analysis, Writing – original draft, Writing – review & editing. LV: Writing – original draft, Writing – review & editing. IH: Writing – original draft, Writing – review & editing. TA: Writing – original draft, Writing – review & editing. LL-C: Writing – original draft, Writing – review & editing. IA: Writing – original draft, Writing – review & editing. ES-E: Writing – original draft, Writing – review & editing. IG-C: Conceptualization, Writing – original draft, Writing – review & editing. VG-G: Writing – original draft, Writing – review & editing. CA: Writing – original draft, Writing – review & editing. PC: Writing – original draft, Writing – review & editing. MO: Writing – original draft, Writing – review & editing. JL-J: Writing – original draft, Writing – review & editing. MAG: Writing – original draft, Writing – review & editing. EA: Writing – original draft, Writing – review & editing. MC-C: Writing – original draft, Writing – review & editing. AA-P: Writing – original draft, Writing – review & editing. CG-S: Writing – original draft, Writing – review & editing. JH-R: Writing – original draft, Writing – review & editing. AR-P: Writing – original draft, Writing – review & editing. MM-C: Writing – original draft, Writing – review & editing. MG: Writing – original draft, Writing – review & editing. JM: Writing – original draft, Writing – review & editing. PR-G: Writing – original draft, Writing – review & editing. JL: Writing – original draft, Writing – review & editing. DC: Writing – original draft, Writing – review & editing. ÁC: Conceptualization, Writing – original draft, Writing – review & editing. CV: Writing – original draft, Writing – review & editing. RM: Conceptualization, Writing – original draft, Writing – review & editing. CS: Writing – original draft, Writing – review & editing.
