# Supplementary material for: Omicron SARS-CoV-2 infection management and outcomes in patients with hematologic disease and recipients of cell therapy
Source: Front Oncol. 2024 Jun 19;14:1389345. doi: 10.3389/fonc.2024.1389345 (PMC11250586; doi:10.3389/fonc.2024.1389345)
Supplement: Supplementary file 1 [file DataSheet_1.docx]

Supplementary Material

# Supplementary Figures and Tables

## Supplementary Table 1

Supplementary Table 1: Commercial PCR test available in participating centers during the study.

| **Commercial COVID-19 detection PCR kit** | **Manufacturer** |
| --- | --- |
| Alinity m SARS-CoV-2 Assay | Abbott Molecular. Illinois, USA |
| Abbott RealTime SARS-CoV-2 Assay | Abbott Molecular, Illinois, USA |
| LightMix® Modular SARS-CoV (COVID-19) | Roche Diagnostics, Pleasanton, USA |
| Aptima® SARS-CoV-2 Assay (Panther® System) | Hologic, Marlborough, Massachusetts, USA |
| TaqPath COVID-19 CE-IVD RT-PCR | Thermofisher Scientific Waltham, Massachusetts, USA |
| SARS-CoV-2 RT-PCR | Vitro, Sevilla, Spain |
| Xpert® Xpress SARS-CoV-2 | Cepheid, Sunnyvale, California, USA |
| SARS-CoV-2 REAL TIME PCR KIT | Vircell, Granada, Spain |

## Supplementary Figure 1

Supplementary Figure 1: (A) Percentage of each type of SARS-CoV-2 variant of concern (VOC) between week 1 of 2021 and week 18 of 2022 (January 4^th^, 2021, to May 8^th^, 2022) among the number of randomly selected sequenced samples. (B) Evolution of Omicron variants between weeks 48 of 2021 and 18 of 2022 (November 29^th^, 2021, to May 8^th^, 2022) and between the number of randomly selected sequenced samples. Available at site:

https://www.sanidad.gob.es/profesionales/saludPublica/ccayes/alertasActual/nCov/documentos/COVID19_Actualizacion_variantes_20220523.pdf
